# Imaging the influence of peripheral TRPV1-signaling on cerebral nociceptive processing applying fMRI-based graph theory in a resiniferatoxin rat model

**DOI:** 10.1371/journal.pone.0266669

**Published:** 2022-04-28

**Authors:** Isabel Wank, Lisa Kutsche, Silke Kreitz, Peter Reeh, Andreas Hess

**Affiliations:** 1 Institute of Experimental and Clinical Pharmacology and Toxicology, Friedrich-Alexander-Universität Erlangen-Nürnberg, Erlangen, Germany; 2 Institute of Physiology and Pathophysiology, Friedrich-Alexander University Erlangen-Nürnberg, Erlangen, Germany; Istituto Italiano di Tecnologia, ITALY

## Abstract

Resiniferatoxin (RTX), an extract from the spurge plant *Euphorbia resinifera*, is a potent agonist of the transient receptor potential cation channel subfamily V member 1 (TRPV1), mainly expressed on peripheral nociceptors—a prerequisite for nociceptive heat perception. Systemic overdosing of RTX can be used to desensitize specifically TRPV1-expressing neurons, and was therefore utilized here to selectively characterize the influence of TRPV1-signaling on central nervous system (CNS) temperature processing. Resting state and CNS temperature processing of male rats were assessed via functional magnetic resonance imaging before and after RTX injection. General linear model-based and graph-theoretical network analyses disentangled the underlying distinct CNS circuitries. At baseline, rats displayed an increase of nociception-related response amplitude and activated brain volume that correlated highly with increasing stimulation temperatures. In contrast, RTX-treated rats showed a clear disruption of thermal nociception, reflected in a missing increase of CNS responses to temperatures above 48°C. Graph-theoretical analyses revealed two distinct brain subnetworks affected by RTX: one subcortical (brainstem, lateral and medial thalamus, hippocampus, basal ganglia and amygdala), and one cortical (primary sensory, motor and association cortices). Resting state analysis revealed first, that peripheral desensitization of TRPV1-expressing neurons did not disrupt the basic resting-state-network of the brain. Second, only at baseline, but not after RTX, noxious stimulation modulated the RS-network in regions associated with memory formation (e.g. hippocampus). Altogether, the combination of whole-brain functional magnetic resonance imaging and RTX-mediated desensitization of TRPV1-signaling provided further detailed insight into cerebral processing of noxious temperatures.

## 1. Introduction

Pain as a vital warning signal (IASP [1]) may evolve into a huge burden for pain patients after chronification and is therefore a research hotspot. Since pain is a multifaceted sensation, there is no single pain area, but a widespread network of brain structures processing its many aspects including stimulus intensity, aversion and saliency [2–5].

Resiniferatoxin (RTX), a naturally occurring diterpene extracted from *Euphorbia resinifera* [6], is an around 100 times more potent agonist of the transient receptor potential cation channel subfamily V member 1 (TRPV1) than capsaicin [7]. TRPV1 is a non-selective cation channel with high Ca^2+^ permeability, mainly expressed on peripheral nociceptive C-fibers [8], and to a lesser extent on Aδ-fibers [9]. Besides, CNS expression of TRPV1 is known e.g. for cortex, hippocampus, amygdala, and periaqueductal grey [10–13]. The channel pore can be opened by protons, endocannabinoids, capsaicin/RTX, and temperatures above 43°C (cell culture). TRPV1 can be sensitized by bradykinin [14] and prostaglandins [15], lowering the activation threshold even to body temperature. Therefore, TRPV1 plays an important role during injury, inflammation, and chronic pain [16, 17], rendering it relevant for analgesic drug development (see [18–22] for review).

Peripheral RTX administration leads to a fast and long-lasting desensitization (> 5 weeks [23]) of dominantly peripheral TRPV1-expressing neurons due to calcium-mediated excitotoxicity and mitochondrial swelling [6, 24–26]. This effect is dose-dependent and partially reversible after months [6, 27]. The resulting block of nociceptive signal transmission from the periphery to the CNS causes lasting analgesia, highly specific for thermal pain [28–30]. The perception of cold, touch-sense, proprioception, and locomotor function remain unaffected [31].

While the action of RTX concerning cellular and peripheral effects is well-described using genetics [23], cell culture [24], *in vitro* preparations [24], and behavioral testing [23], the knowledge about how desensitization of TRPV1-signaling impacts thermal CNS nociception is minimal.

Functional magnetic resonance imaging (fMRI) is noninvasive, reliable and therefore gold-standard to unravel brain functions in wellbeing and disease. Consequently, peripheral application of RTX in combination with stimulus-driven and resting state (RS) fMRI could shed new light on how the brain responds selectively to innocuous and noxious thermal input from the periphery.

Our major aims, also achieved within the scope of this study, were a) to distinguish CNS responses for warmth and nociceptive heat by RTX-mediated TRPV1-desensitization, b) to reveal how graph-theoretical analyses depict changes in brain function that cannot be identified by classic GLM-based analyses and c) to investigate if desensitization of TRPV1-expressing fibers impacts on CNS resting-state networks.

## 2. Material and methods

### 2.1 Animals

The study was performed on male Wistar IGS rats (350–500 g, average weight 420 g, age: 14 weeks) purchased from Charles River, Sulzfeld, Germany. All animals were housed in groups of five at the animal facility of the Institute of Pharmacology and Toxicology, University of Erlangen-Nuremberg at a 12 h-day-night-cycle with food (Altromin, Lage, Germany) and water supplied *ad libitum*.

Only male rats were used to limit animal numbers.

All experiments were conducted according to the guidelines of the Federation of European Laboratory Animal Science Association (FELASA) and were approved by the local ethical committee for research animal care (government of Unterfranken) under license 55.2.2–2532.2–957.

### 2.2 Experimental design, endpoints and resiniferatoxin injections

**Fig 1:**
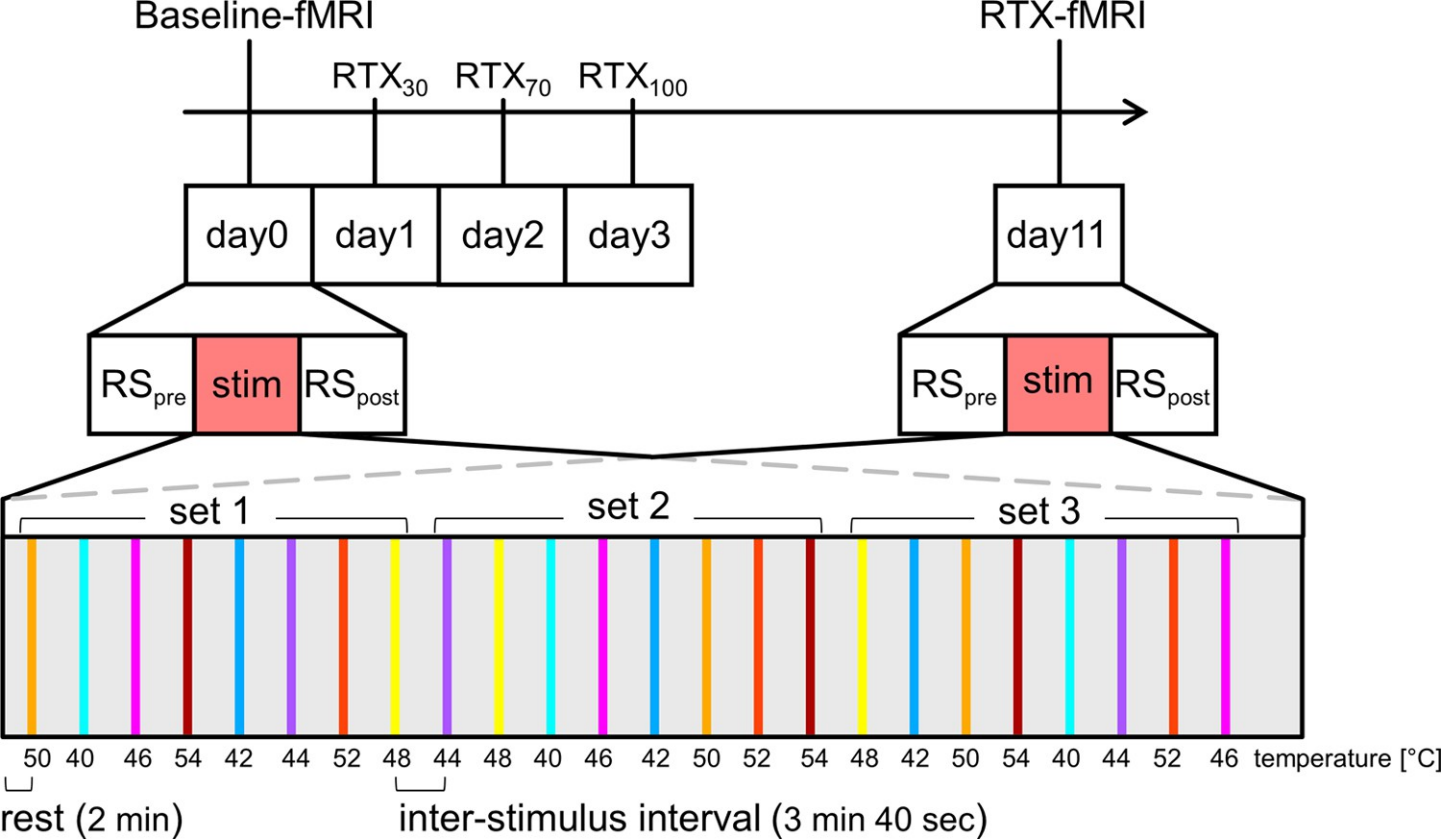
Experimental outline. Following a first fMRI scan at day 0 (baseline), animals were injected s.c. with increasing doses (30, 70, 100 μg/kg) of RTX on three consecutive days. Eight days following the last injection, a second fMRI scan (RTX) was performed, to assess the effects of desensitization of TRPV1-expressing neurons on central temperature processing. Each fMRI scan included two resting state sessions, one before (RS_pre_) and one after (RS_post_) the thermal stimulation. During the stimulation period, the contact heat stimuli sequence was presented at the dorsal side of the left hind paw of the animal. Temperatures ranging from 40°C to 54°C were arranged in a pseudo-randomized fashion per set, and were held for 20 sec each followed by 3 min 40 sec rest. Three different sets were presented during the 100 min scan. This protocol was re-used for every animal.

The experiment was designed as a within-animal before-after-drug-comparison: naïve animals (baseline, n = 18) were measured once for assessing baseline using resting state (RS) and stimulus-driven fMRI (Fig 1). The next day, RTX solution (Sigma-Aldrich, St. Louis, USA) was prepared according to the protocol of Mitchell *et al*. [32], and injected s.c. into the neck skin on three consecutive days with increasing doses (30, 70, 100 μg/kg) to desensitize TRPV1-expressing fibers. All injections were performed under isoflurane anesthesia, as injection of RTX evokes a temporary burning pain due to massive stimulation of the TRPV1-receptor, which is described by humans as mild to moderate [33]. As the TRPV1-expressing neurons desensitize very quickly and permanently, short anesthesia, which is also the protocol for intra-articular RTX injection in arthritic dogs [34], should be sufficient to prevent pain and stress for the rats. To avoid shock reactions and respiratory failure due to bronchoconstriction and enhanced mucus secretion [35], the daily dose was split into two injections (separated by at least 5 h). Additionally, a protective mixture of terbutaline sulfate (0.4 mg/kg; Astra Zeneca, London, UK), atropine sulfate (0.8 g/kg; Dr. F. Köhler Chemie, Bensheim, Germany) and theophylline (32 mg/kg; Riemser Pharma, Greifswald, Germany) was injected shortly before each RTX injection to prevent respiratory stress [36]. Animals were monitored closely after RTX injection for at least 4 h. Supportive oxygen was applied if the animals showed breathing distress.

Humane endpoints were used to prevent excessive distress: possible burden of the animals was assessed daily by trained personnel using a specific scoresheet. If burden occurred, animals were assessed twice a day. If no burden was noticed, the animals were weighed twice a week, otherwise daily. Final endpoints comprised dehydration, reduction of body weight > 20% from baseline, severe circulation problems (cyanosis, shivering, cold to the touch), convulsions, automutilation, lesions of the thermo-stimulated hind paw (see Section 3.4), sickness behavior (labored breathing, ruffled fur, hunched posture, reduced social interaction such as separation from cage mates, reduced reaction to stimuli), infection of injection sites (abscesses, necrosis), and persistent breathing problems after RTX injection for more than three hours. Five rats met final endpoints due to persistent breathing problems after RTX injection and were euthanized *ad hoc* using an overdose of CO_2_. Cardiovascular arrest was confirmed to ensure death.

With all remaining animals, a second fMRI experiment was performed eight days after the last injection, to ensure a maximal treatment effect. Measurements of four RTX animals did either not meet quality criteria (one animal with less than 0.5% overall blood oxygenation level dependent (BOLD) signal change and motion artifacts during the measurement) or had technical problems (stimulation failures), and had to be excluded from further analysis (RTX, n = 9). Due to the relatively high treatment-associated dropout, we refrained from restocking the animal number only to reach equal numbers for the sake of the 3R animal welfare principle. The strong and robust findings of the RTX-effect made us confident to achieve sufficient statistical power (see Section 4.5 Statistical analysis). On note, due to uneven animal numbers in baseline and RTX group, no paired statistical testing could be performed.

## 3. Functional MRI procedures

### 3.1 Animal preparation and MR-hardware

Rats were anesthetized with 5% isoflurane (cp-pharma, Burgdorf, Germany) in medical air for 4 min. Next, the animals were transferred onto a specially designed acrylic glass cradle (Bruker BioSpin MRI GmbH, Ettlingen, Germany) with an integrated water heating system to maintain body temperature stable at 37°C. The head of the animal was firmly fixed atraumatic by the front teeth in a nose-mouth-mask to minimize motion artifacts. Low doses of isoflurane (~1.25%) were supplied directly into this mask to maintain anesthesia for the whole duration of the fMRI experiment. Breathing rate of the animals was monitored all the time using a pediatric breathing sensor and a dedicated animal monitoring device. The depth of anesthesia was adjusted to achieve a constant breathing rate between 60–65 breaths/min, ensuring maximal BOLD contrast and minimal head movement [37]. The eyes were covered with an eye and nose ointment (Bepanthen, Bayer Vital GmbH, Leverkusen, Germany) to prevent exsiccation damage.

FMRI measurements were performed on a 4.7 T small animal MRT (Bruker Biospec, Bruker BioSpin MRI GmbH, Ettlingen, Germany) operated under ParaVision software V. 5.1 (Bruker Biospec; Bruker BioSpin MRI GmbH, Ettlingen, Germany). The scanner was equipped with a 200 mT/m gradient system and a RF-coil system for excitation. The actively decoupled 2x2 rat array head coil (Rapid Biomedical GmbH, Rimpar) used for signal detection ensured good signal-to-noise-ratio.

### 3.2 MRI preparation and protocols

An initial scout measurement with three orthogonal slices was conducted to achieve ideal positioning of the animal in the scanner and to ensure good coil positioning. A fast gradient echo single shot Echo Planar Imaging (GE EPI) sequence (TR = 200 ms; TEeff = 25.3 ms; field of view (FOV) 25 * 25 mm; slice thickness 1 mm; matrix 64*64 voxel) was used to record 300 repetitions of one axial slice positioned in the caudal part of the brain. Played as a movie, this allowed detecting head motion of the rat due to insufficient fixation. If motion of more than one pixel was detected, the fixation of the animal was adjusted. One volume of 22 axial slices spanning the brain from Bregma -15.72 mm to 6.12 mm with an in-plane resolution of 0.391 x 0.391 mm was acquired using GE EPI (TR = 2000 ms; TEef = 25.3 ms; FOV 25 * 25 mm; slice thickness 1 mm; matrix 64 * 64 voxel) to reassure good image quality. Only if inevitable, local field inhomogeneities were corrected using the FastmapScout [38] macro (semi-automatic preset for localized 1^st^ and 2^nd^ order shimming) implemented in ParaVision 5.1.

### 3.3 Resting state fMRI

After finishing the scanner settings, a 10 min resting state fMRI was acquired each time before (RS_pre_) and after (RS_post_) the stimulus-driven fMRI sequence (Fig 1) using GE EPI (TR = 2000 ms; TEef = 25.3 ms; FOV 25 * 25 mm; voxel size 0.391 * 0.391 mm; slice thickness 1 mm; matrix 64 * 64 voxel, 22 axial slices also covering the brain from bregma -15.72 mm to 6.12 mm, 300 repetitions).

### 3.4 FMRI stimulation paradigm

To investigate differences in warmth and nociceptive processing, pseudo-randomized thermal stimuli ranging between 40°C to 54°C target temperature at intervals of 2°C (yielding eight different stimuli in total) were applied at the dorsal side of the left hind paw of the animal at specific time points. This temperature range covers the whole activation temperature range of the TRPV channels. Stimuli were applied through an actively feedback computer-controlled Peltier heating device (developed in-house) with no interference from and to the MRI scanner allowing to achieve precisely the stimulation temperatures ± 1°C. Baseline Peltier temperature at rest was 33.5±1.3°C.

Each fMRI measurement started with a two min rest period, to allow the animal to adapt to the background noise of the scanner. Thermal stimuli were held for 20 seconds each (5 sec ramp, 15 sec plateau), followed by 3 min 40 sec rest respectively, to ensure cool-down of the paw and to let the brain activity fully return back to baseline. One set consisted of all eight stimuli arranged in a pseudo-randomized manner to prevent habituation and anticipation of the animal. All temperature stimuli were presented in three different consecutive sets to obtain reliable data for GLM (general linear model) analysis. Double presentation of the same temperature at the transition of successive sets was avoided. This stimulation protocol was re-used for every animal (Fig 1). During presentation of the three sets of those eight thermal stimuli, 3000 functional BOLD-weighted gradient echo single shot EPI scans with the same parameters as stated above for resting state were acquired. The whole stimulus-driven fMRI experiment lasted 100 min.

### 3.5 Anatomical MRI and follow-up care

Finally, 22 corresponding anatomical T2 reference images (RARE [39]; TR = 3000 ms, TEef = 47.1 ms, RARE factor 8, FOV 25 * 25 mm, slice thickness 1 mm, matrix 256 * 256 voxel, 22 axial slices) were taken at identical positions as the functional images for anatomical reference. After finalizing the measurement, the animal was removed from the scanner and the stimulated hind paw was treated with cooling foam spray containing panthenol (Bepanthen, Bayer Vital GmbH, Leverkusen, Germany). The animal was placed in an empty box with tissue-covered bedding until it was fully recovered and finally returned to its home cage.

## 4. FMRI analysis

Functional data analyses of RS and stimulus-driven fMRI data were performed using BrainVoyager QX (Version 2.8.0; Brain Innovation BV, Maastricht, Netherlands) and MagnAn (BioCom GbR, Uttenreuth, Germany) as described previously [40, 41]. For graph-theoretical analyses, we also used algorithms implemented into the NetworkWorkbench toolbox (Version 1.0.0) [42]. Visualization was done in Amira (Version 5.4.2, Thermo Fisher Scientific Inc., Waltham, USA).

### 4.1 Stimulus-driven fMRI analysis

#### 4.1.1 Preprocessing of stimulus-driven BOLD fMRI & GLM analysis

Every two consecutive volumes were averaged to reduce noise, resulting in a TR_eff_ of 4000 ms. After discarding the first two volumes of the datasets to avoid MR saturation effects, preprocessing [scan time correction (ascending interleaved, interpolation method cubic spline), motion correction to eliminate the minimal rat head movement (registration to first brain volume; trilinear detection and sinc interpolation), spatial (Gaussian smoothing with kernel size of 2 pixel) and temporal smoothing (linear and non-linear high pass filtering, kernel 12s FWHM, FFT 9 cycles)] was performed for each animal separately in BrainVoyager QX. After preprocessing, a first order GLM analysis with separate predictors for each stimulation temperature was performed voxel-wise to calculate correlation between the time courses and the stimulation protocol (folded by a rat-specific two gamma HRF implemented in BrainVoyager QX using the following parameters: onset 0, time to response peak 16 s, response dispersion 1, response undershoot ratio 1, time to undershoot peak 40 s, undershoot dispersion 1).

Consequently, separate statistical parametric maps (SPMs) were obtained for each animal and for each stimulation temperature.

#### 4.1.2 Group analysis of stimulus-driven BOLD fMRI

For the second order group analysis, the obtained SPMs of all animals had to be registered using a workflow well established [41, 43, 44]. Therefore, based on anatomical grey values, a spatial registration to a center-positioned reference animal in terms of an affine registration with 6 degrees of freedom (translation x-y-z, scaling x-y, rotation z) was applied using MagnAn. The thereby obtained registration matrix was then applied to the SPMs, yielding center-positioned well-aligned SPMs.

For each animal, the SPMs (separate for each temperature) were corrected for multiple comparisons (false discovery rate FDR, q = 0.05, confined by brain mask) and the resulting significantly activated voxels were identified as belonging to one out of 202 separable brain structures using an adapted 3D Paxinos rat brain atlas aligned to the SPM-corresponding anatomical grey values [45]: 22 atlas slices that corresponded best to our fMRI slices were chosen manually from the Paxinos rat brain atlas. They were converted into a digital rat brain atlas, where each brain structure (for left and right hemisphere separately) had a unique index number and coloring assigned. The atlas slices were adjusted to fit best to an average of the pre-registered center-positioned grey value single animal anatomical fMRIs (as described above) and converted into structure-specific ROIs. Using the single animal registration matrix, the ROIs were then inversely transformed back into subject space.

Each registration step, of grey value anatomical fMRI data as well as the inverse transformation of the ROIs, was inspected very thoroughly and—if necessary- adjusted manually. The quality control was performed iteratively and with great attention to detail, until the results were satisfactory.

Applying the atlas in subject space also to the FDR-corrected activation maps, the number of activated voxels per brain structure was obtained per animal and averaged group-wise. This mean number of activated voxels per brain structure, defined as the activated brain volume, was calculated for each stimulation temperature and experimental group independently.

Next, animal-wise event-related mean BOLD signal time courses were calculated from all these significantly activated voxels of each brain structure, spanning from 10 time points before to 10 after stimulation period. Using these time courses, the corresponding response amplitude per brain structure was calculated again per animal for each stimulation temperature, as well as averaged for each stimulation temperature and experimental group. This approach allowed comparing these groups directly via quantifiable parameters. For comparison of the spatial distribution of the maximal BOLD response amplitude, their maximum was calculated voxel-wise for each animal and stimulation temperature. Those maximum BOLD amplitude maps were registered using the same parametrization/registration matrix (see above) and also averaged per group.

#### 4.1.3 Graph-theoretical analysis of stimulus-driven BOLD fMRI

As already described in [43], the interaction between all activated brain structures can be assessed via graph-theoretical network analyses, based on the temporal correlation of the structure-specific mean time courses obtained in the previously described step.

First, a global regression analysis (removal of the global mean) was performed to exclude the strong global response to the stimulation. Second, a full adjacency matrix was generated calculating the Pearson correlation coefficient between mean time courses of all activated brain structures per predictor (= stimulation temperature) and per animal. After transformation of the correlation coefficients into Fisher-z-values to ensure Gaussian distribution, a mean adjacency matrix was calculated for each stimulation temperature and experimental group (baseline and RTX). To achieve an optimal topological comparability, matrices were standardized to contain the same number of connections between nodes, using the standard k-value of 10, resulting in the 1010 strongest connections (202 brain structures, theoretically 10 edges per node, symmetric). Only positive correlations were included in the further analysis (see [46] for explanation). Network topology of both groups was compared regarding the normalized shortest path length λ, normalized clustering coefficient γ and the resulting small world index σ [47]. Blondel communities [48] (NetworkWorkbench toolbox)–allowing to structure large networks by detecting and grouping brain regions with high interaction–were calculated and visualized in 2D applying Kamada-Kawai-projections.

### 4.2 Resting state fMRI analysis

#### 4.2.1 Preprocessing of resting state BOLD fMRI

Similar to the stimulus-driven data sets, the first two volumes were discarded due to saturation effects. Preprocessing in BrainVoyager QX comprised only the scan time correction (ascending interleaved, interpolation method cubic spline) and motion correction (registration to first brain volume; trilinear detection and sinc interpolation). All further analyses, including preprocessing with a FFT low pass filtering at 0.1 Hz, were conducted in MagnAn.

#### 4.2.2 Graph-theoretical group analysis of resting state BOLD fMRI

As described above for the stimulus-driven fMRI, animals were registered to the same reference animal and the atlas brain region labels were allocated as described above. Individual brain masks were used to confine the analysis to voxels inside the brain. A seed region (1.173 mm diameter) was placed at the center of mass of each brain region for multi-seed region analysis [49]. The Pearson correlation coefficient *r* was calculated between the average time course of each seed region and all remaining single voxels within the brain. This approach yielded one correlation volume per seed region for each animal in the individual animal space. Significant correlations were determined for each correlation volume using false discovery rate (q = 0.05). After converting *r*-values to Fisher-z-values, the correlation values of all voxels belonging to the same brain region were averaged. For each seed, this yielded one row in a new asymmetric connectivity matrix, summarizing the correlation between these seed regions and the remaining brain structures.

Again, the matrices were standardized to k = 10, containing only the 2020 strongest positive connections (202 brain structures, directed).

### 4.3 Graph-theoretical visualization

Correlation matrices were displayed as 3D brain networks, with nodes representing the color-coded brain regions at their anatomical 3D/2D positions (center of gravity) and the edges between the nodes representing the correlation coefficient (functional connectivity, FC). Size of nodes and thickness of edges represent number of connections (degree) and the weight of the correlation coefficient, respectively.

To allow for a more condensed representation, the independently calculated 202 brain structures were merged according to the functional group they belong to, e.g. all structures of the sensory cortex were merged together into the functional group ‘sensory cortex’. All changes in degree (number of connections one structure has to others) or FC were included, to obtain one value for each functional group / each connection. After statistical comparison (see Methods 4.5.), edges were color-coded to visualize if FC was increased (red) or decreased (blue).

As structures belonging to the same functional group may appear in separate Blondel communities (e.g. somatosensory cortices are often split into two communities representing each hemisphere), only structures within the same community were summed up to sustain that information. Therefore, nodes representing the same functional group (sensory cortex) may appear in different communities (e.g. two communities representing the cortical hemispheres, each containing one summed up node for all somatosensory cortices in this community).

### 4.4 Blinding

The fMRI analyst was not blinded. Instead, all animals were batch processed in one standardized analysis workflow at the same time. The workflow allows no animal-specific input that may be misused to generate bias.

### 4.5 Statistical analysis

**Fig 2:**
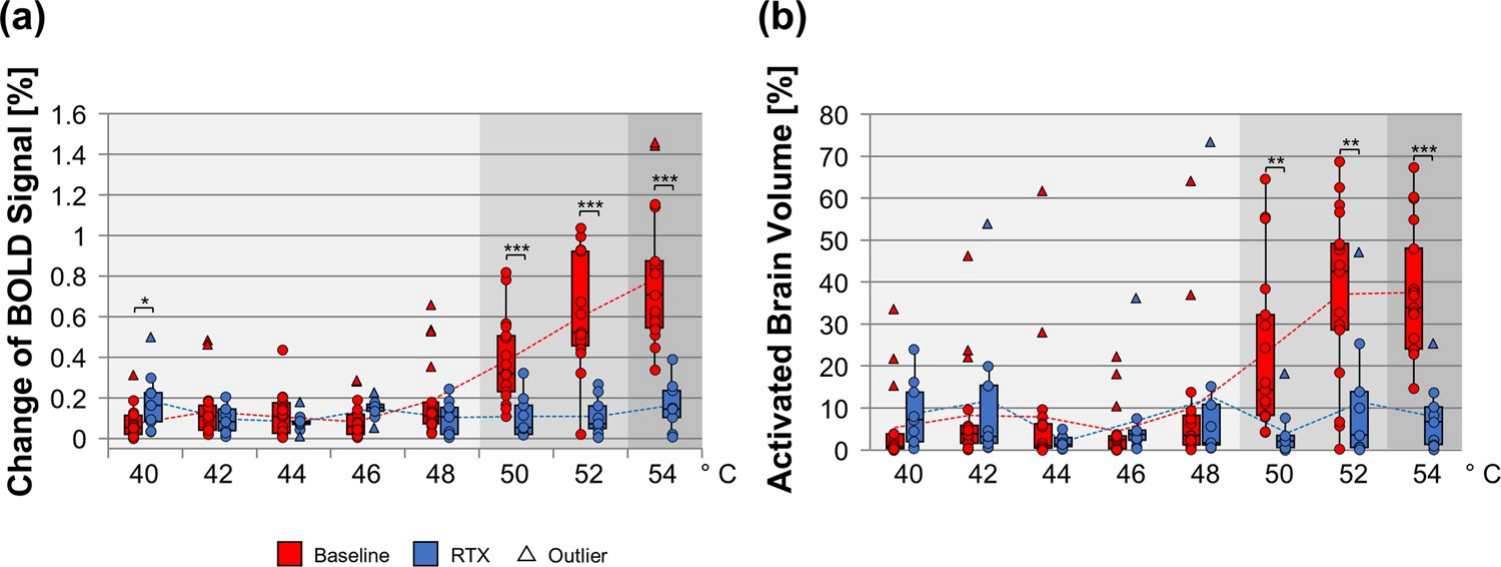
Whole brain BOLD response amplitude and activated brain volume evoked by different temperatures. Depicted are single animal values (dots) as well as mean (dotted lines) BOLD response amplitude (a) and activated volume (b) over all brain areas evoked by innocuous (light grey box), noxious (middle grey box) and highly noxious (dark grey box) temperatures for both groups. Whereas the untreated animals (baseline, red) showed increasing brain responses well correlated to the linear temperature increase (r_Amplitude_ = 0.89, r_Volume_ = 0.88; also see S1 Fig, correlation coefficient), RTX-treated rats (blue) failed to show increased responses to temperatures between 48°C and 52°C (r_Amplitude_ = -0.04, r_Volume_ = 0.03). With 54°C, a slight but insignificant (p_52vs54°C_ = 0.067, paired Student’s t-test) increase could be noted for the response amplitude. Activated brain volume is represented as percentage of total brain volume per animal. Statistical significance between groups was calculated using homoscedastic Student’s t-test and corrected for multiple comparisons by FDR q = 0.05. Boxplots span the 25% to the 75% quartile (median included). A horizontal line marks the median. The whiskers represent minimum and maximum values. Values below 1.5 times the interquartile range from minimum and above 1.5 times the interquartile range from maximum are considered as outliers (triangles). Means of each group are connected by a dotted line. * p≤0.05; ** p≤ 0.01; *** p≤ 0.001. (n_Baseline_ = 18; n_RTX_ = 9).

We are aware of the large dropout of the RTX group. But the RTX effects described here are highly significant even with the low number of 9 rats, and profound RTX-mediated reduction in BOLD signal can even be found on subject-level in every single animal (see Fig 2). Therefore, we are confident that our statistics provide sufficient power to draw conclusions.

If not displayed as single-animal values, BOLD parameter data (BOLD response amplitude and activated brain volume) are presented as mean ± SEM. In advance, one-factor-ANOVA with the eight stimulation temperatures as factors was performed to analyze group-wise differences between the BOLD response amplitudes. Tukey HSD post-hoc test was used to assess statistical significance and correct for multiple comparisons.

Mean group values were compared by homoscedastic two-tailed Student’s t-test to assess significant differences between both groups. All tests were corrected for multiple comparison issues using Benjamini-Hochberg FDR method at q = 0.05. If temperature-associated values were compared within groups, a paired Student’s t-test was used. Statistics were calculated in Excel (Version 2016; Microsoft, Albuquerque, USA).

For second order statistics, the maximum BOLD amplitude maps were averaged group-wise separately for each stimulation temperature. Significant differences between the groups were calculated voxel-wise using homoscedastic Student’s t-test and controlled for the family-wise error with permutation testing using 1000 random permutations [50]. P-values below 0.05 were accepted as significant. This threshold was chosen to cover also the weaker differences found for low temperatures. Second order statistics were performed in MagnAn.

Regarding the stimulus-driven brain networks, significant differences in functional connectivity (i.e. reduction or enhancement of correlation) between baseline and RTX were assessed for each stimulation temperature applying network based statistics (NBS) based on Zalesky *et al*. [51] implemented in MagnAn: a homoscedastic Student’s t-test was calculated for each temperature between the mean matrices of both experimental groups and limited to α = 0.05. The size of the biggest component (number of interconnected nodes) was determined (number of connections). Using 1000 permutations, animals were randomly assigned to each group, and t-tests were recalculated. The number of t-tests yielding bigger components than the original comparison was noted, and a new p-value was calculated by dividing this number by 1000. If this new p-value was below 0.05, the differences found in the original comparison were accepted as statistically significant. Differences with a p-value ≤ 0.05 were only found for the temperatures 50°C, 52°C, and 54°C.

Differences in small world parameters between baseline and RTX were calculated in Excel by homoscedastic two-tailed Student’s t-test and were corrected for multiple comparison issues using Benjamini-Hochberg FDR method at q = 0.05.

To assess fundamental differences in RS-networks, the resting state condition before the stimulus-driven fMRI (RS_pre_) was compared between baseline and RTX using NBS as described above.

For statistical comparison of the RS-networks before (RS_pre_) and after (RS_post_) the stimulus-driven fMRI, a different approach had to be used due to restrictions concerning the ‘control group’ in paired NBS method. For NBS, no change in control group conditions is presumed. Here, resting state networks before and after the heat stimulation had to be compared. As it is known that heat stimulation influences the resting state networks [52], the control group (baseline) did not meet those requirements. Therefore, statistical differences between RS_pre_ and RS_post_ were calculated using a paired two-tailed Student’s t-test in MagnAn, and were permutation-controlled for the family-wise error. Resulting p-values ≤ 0.05 were accepted as significantly different.

## 5. Results

### 5.1 Classical BOLD signal analysis

To investigate the impact of the RTX-treatment (RTX), the average whole brain activation level (BOLD response amplitude, Fig 2A) and the activated brain volume (i.e. the number of voxels activated displayed as percentage of total brain volume, Fig 2B) was compared between baseline and RTX. In advance, a one-factor-ANOVA with the eight stimulation temperatures as factors was performed per group. At baseline, significant differences (p<0.05, Tukey HSD post-hoc test) were found between temperatures ≤ 46°C and those ≥ 50°C, as well as between 48°C and ≥ 52°C, and between 50°C and ≥ 52°C.

For RTX, no significant differences at all were found between the stimulation temperatures.

On the whole brain level, the baseline data showed a steep increase of BOLD response amplitude and activated brain volume that correlated positively with the linear temperature rise beginning with 48°C (Fig 2 and S1 Fig; r_Amplitude_ = 0.89, *r*_*Volume*_ *= 0*.*88*). Below 48°C, activation level was minimal and comparable between both groups. RTX showed no increase with rising temperatures (RTX: r_Amplitude_ = -0.04, r_Volume_ = 0.03) except a slight but statistically insignificant increase of the amplitude at 54°C (p_52vs54°C_ = 0.067). The differences between baseline and RTX were found to be highly significant for all temperature above 48°C (p_Amplitude_ ≤ 0.0008; p_Volume_ ≤ 0.008).

**Fig 3:**
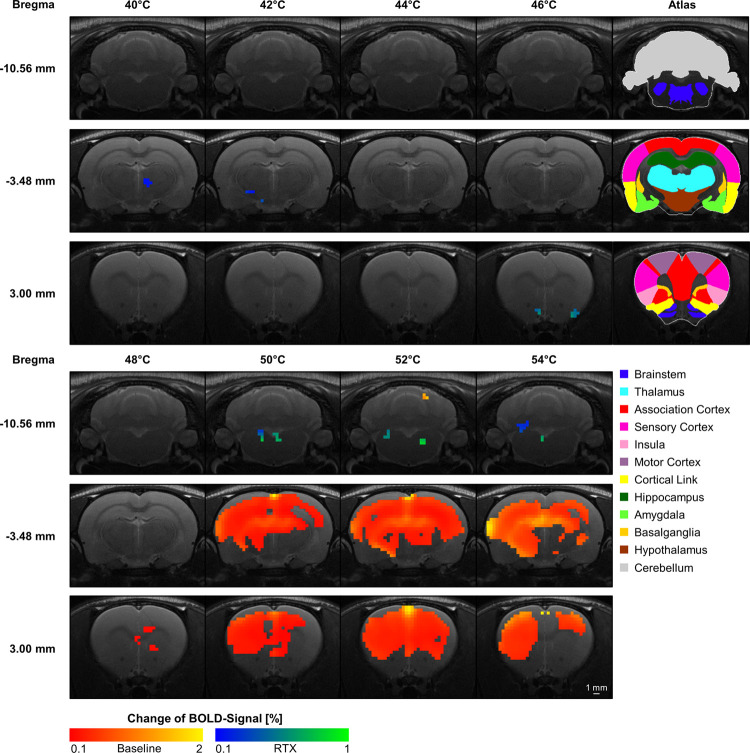
Significant differences in the whole brain maximal BOLD response amplitude evoked by different temperatures before and after RTX-treatment. The voxels in red-yellow showed greater activation at baseline, whereas the blue-green voxels showed greater activation after RTX treatment. Only very small differences (raw data showed comparable activation for both groups, data not shown) could be shown for temperatures ≤ 48°C, indicating that processing of innocuous temperatures remained unaffected by RTX and the resulting desensitization of TRPV1-expressing neurons. However, baseline showed significantly higher activation for noxious temperature above 48°C in wide areas of the brain (subcortical and cortical). Data are represented as percentage change of maximal BOLD response amplitude. Only voxels with p-values ≤ 0.05 (FWE permutation corrected) are shown. (n_Baseline_ = 18; n_RTX_ = 9).

To assess where those differences were spatially located within the brain, the maximal BOLD response amplitude of each animal, averaged for each treatment were compared voxel-wise between baseline and RTX for each temperature (Fig 3).

For the temperatures from 40°C up to 48°C, almost no differences in the maximal BOLD response amplitude could be found. Concerning the clearly noxious temperatures ranging from 50°C up to 54°C, significant and widespread differences could be found between baseline and RTX: baseline (red-yellow voxels) showed significantly higher activation levels in great parts of subcortical and cortical brain regions, with the exception of some parts of piriform cortex, medial thalamus, hypothalamus and the brainstem. A closer look into the single brain structures activation level of the brainstem revealed that solely the gigantocellular reticular nucleus, the pons, raphe nuclei and olfactory nucleus showed a greater activation level in the RTX group (Fig 3, blue-green voxels).

**Fig 4:**
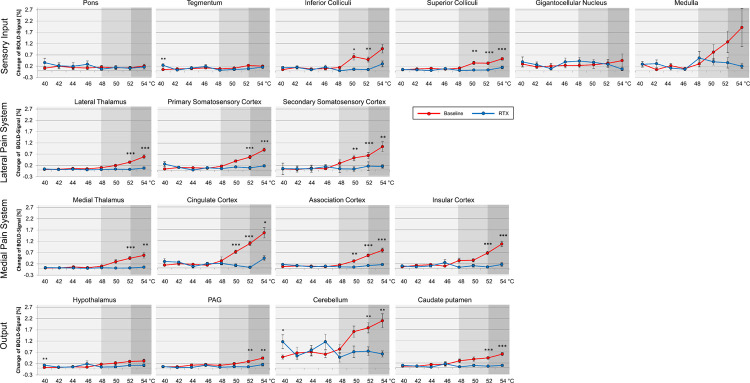
Mean BOLD response amplitudes for representative functional groups. Desensitization of TRPV1-expressing neurons (RTX) had a widespread effect on both sensory-discriminative and emotional-affective brain regions, as well as on some output structures. No or only minor differences between groups were found for brain structures not involved in stimulus-driven tasks, such as the pons, the tegmentum and the output structure hypothalamus, which instead showed significant differences for the activated brain volume, indicating that many voxels were activated at baseline, but with low amplitude (S2 Fig). Statistical significance between groups was calculated using homoscedastic Student’s t-test and corrected for multiple comparisons by FDR q = 0.05. Data are represented as mean ± standard error (SEM). * p≤0.05; ** p≤0.01; *** p≤0.001. (n_Baseline_ = 18; n_RTX_ = 9).

To further break down the results of the whole brain maximal BOLD response amplitude (Fig 3), the mean BOLD response amplitude (Fig 4) and activated volume (S2 Fig) were calculated for each brain region identified via the customized Paxinos rat brain atlas, and were next averaged for functionally distinct subgroups of brain structures (functional groups). The chosen structures represent in great parts the brain regions formerly described as ’pain matrix’ [3, 53]: pain as a complex and multilayered perception is not processed by a single brain structure but by the interaction of a vast network of many brain structures (S3 Fig) in two major streams, the sensory-discriminative lateral and the emotional-affective medial pain pathway [54]. This includes the lateral and medial thalamus, primary (S1) and secondary somatosensory cortex (S2), cingulate (Cg), insular (Ins) and association cortex (Ass), hippocampus (HC), amygdala (Am), structures of the basal ganglia (BG, mainly the caudate putamen and the nucleus accumbens), hypothalamus (Hy), periaqueductal grey (PAG) and the motor output structures (motor cortex (M) and cerebellum (Cer)).

#### 5.1.1 Sensory input: The brainstem

Part of the brainstem structures such as the pons, tegmentum and the gigantocellular nucleus showed no increased activation (BOLD response amplitude) above 48°C for baseline and RTX. At baseline, the medulla responded almost in form of a linear increase to temperatures above 48°C, as the rats increased their breathing rate according to their temperature-dependent stress level (from baseline 60 breaths/min up to 80–90 breaths/min during high temperature stimulation). For temperatures below 48°C, the stimulus-evoked activity in RTX-treated animals was comparable to baseline.

At baseline, both, the inferior and the superior colliculi, showed a significant increase in activation for temperatures above 48°C, whereas RTX only showed a slight (inferior colliculi: p_52vs54°C_ = 0.113, superior colliculi: p_52vs54°C_ = 0.0397) increase at 54°C.

Next, the effects of the desensitization of TRPV1-expressing neurons on the lateral and medial pain system were assessed, the first being involved in the sensory-discriminative processing of nociception, the latter one in the emotional-affective component [5].

#### 5.1.2 Lateral pain system: Lateral thalamus and sensory cortices

Beginning with the lateral thalamus, almost no activation was found after RTX even with the highest temperature, whereas baseline showed a linear increase (Pearson *r*_*Baseline*_ = 0.88, *r*_*RTX*_ = 0.41, S1 Fig) in activity starting with a stimulation temperature of 50°C. The primary (*r*_*Baseline*_ = 0.89, *r*_*RTX*_ = -0.1) and secondary somatosensory cortex (*r*_*Baseline*_ = 0.92, *r*_*RTX*_ = 0.6) followed this scheme. The secondary somatosensory cortex showed greater sensitivity reacting already to 48°C at baseline (p_46vs48°C_ = 0.056).

In summary, no increase of activation was found after RTX within the structures of the lateral pain system, even with the highest temperatures.

#### 5.1.3 Medial pain system: Medial thalamus, cingulate, association and insular cortex

The level of activation of the medial thalamus was nearly identical to that of the lateral thalamus at baseline and after RTX (*r*_*Baseline*_ = 0.91, *r*_*RTX*_ = 0.17), indicating again no significant increase of activation after RTX. The stimulus-response curve of the cingulate cortex (*r*_*Baseline*_ = 0.89, *r*_*RTX*_ = 0.01), insular cortex (*r*_*Baseline*_ = 0.88, *r*_*RTX*_ = 0.11) and the association cortex (*r*_*Baseline*_ = 0.87, *r*_*RTX*_ = -0.05) of both groups also followed this pattern. Cingulate (p_52vs54°C_ = 0.037) and insular cortex (p_52vs54°C_ = 0.046) exhibited a significant increase in activation at the highest temperature of 54°C after RTX. This reaction was most pronounced in the cingulate cortex.

#### 5.1.4 Limbic and motor output: Hypothalamus, periaqueductal grey, caudate putamen and cerebellum

No significant difference in the response of the hypothalamus was found between both groups for temperatures above 48°C, but correlation of the stimulus-response curve was greater at baseline (*r*_*Baseline*_ = 0.95, *r*_*RTX*_ = 0.14; S1 Fig). The PAG responded to temperatures above 48°C for baseline, but only to 54°C after RTX (p_52vs54°C_ = 0.035) (*r*_*Baseline*_ = 0.9, *r*_*RTX*_ = 0.48). The cerebellum showed a huge increase in activation above 48°C for baseline and no response after RTX (*r*_*Baseline*_ = 0.92, *r*_*RTX*_ = -0.4).

### 5.2 Brain network efficacy

**Fig 5:**
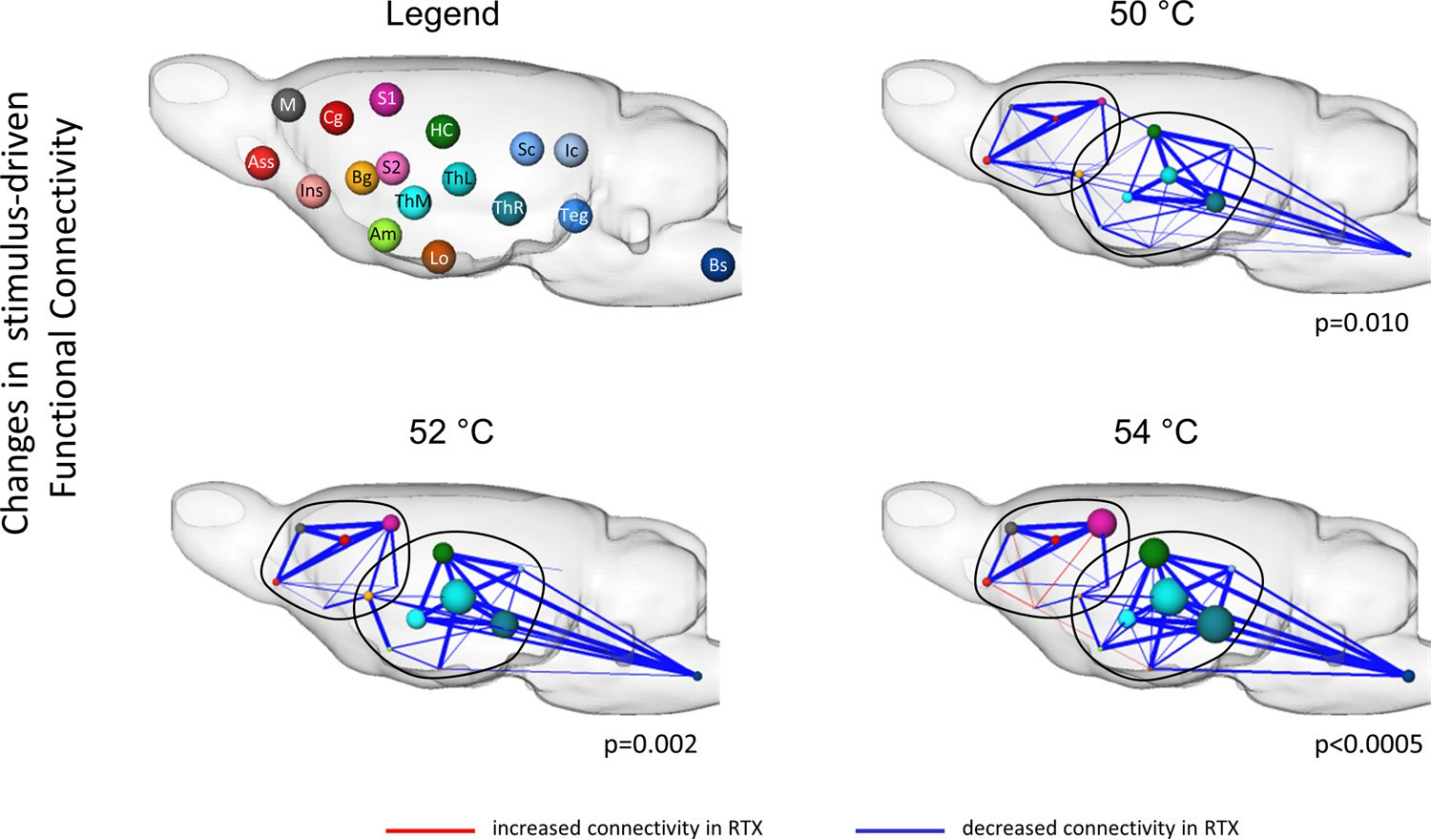
Administration of RTX led to a widespread reduction of stimulus-driven functional connectivity in two distinct brain networks serving for noxious temperatures. Direct comparison of baseline and RTX revealed that two distinct subnetworks (encircled) of brain structures described below showed significantly decreased (blue edges) FC after RTX administration. While thalamo-cortical and hippocampal-cortical connections remained intact (no change), a great reduction was found between thalamus, hippocampus and brainstem such as the superior colliculi and, independently, within cortical structures (primary and sensory somatosensory cortex, association cortex and cingulate cortex). Different functional groups are distinguished by the node colors. Node size represents number of significantly different connections each node has to the others. Thickness of edges represents strength of connection (sum of Pearson r of all significantly different connections). Statistical significance was calculated using NBS, thereby correcting the p-value. (n_Baseline_ = 18; n_RTX_ = 9). Abbreviations: Am: amygdala; Ass: association cortex; BG: basalganglia (esp. striatum); Bs: brainstem structures; Cg: cingulate cortex; HC: hippocampus; Ic: inferior colliculi; Ins: insular cortex; Lo: limbic output/hypothalamus; M: motor cortex; S1/S2: primary/secondary somatosensory cortex; Sc: superior colliculi; Teg: Tegmentum; thM/L: medial/lateral thalamus; ThR: other thalamic nuclei.

The findings from classical BOLD-signal analysis hinted at a disruption of the thalamic input (due to desensitization of the TRPV1-expressing neurons) leading to a very profound suppression of almost the whole brain activation. From the following graph-theoretical analyses, we expected to gain more detailed information how the interaction of single brain regions changed after RTX. Therefore, we investigated next the functional connectivity (FC) in different brain networks first at a global efficacy level (S4 Fig) and second by detailed network analysis showing changes in FC within the functional groups (Fig 5).

No change over the applied temperature range could be found for either group concerning the normalized cluster-coefficient (S4A Fig). A slight increase with rising temperatures could be found for the normalized path length (S4B Fig). Besides 40°C, no significant differences could be found between both groups for any parameter. Regarding the small world-index (relation between normalized cluster-coefficient and normalized path length) as a measure of global network information flow efficacy, only a slight and insignificant decline could be found for temperatures above 50°C, which resembled a decreased efficiency in information processing of noxious temperatures (S4C Fig). This effect is mainly based on the increasing path length and frequently found for noxious thermal stimulation, as networks are usually denser. It was comparable between both groups and is therefore most likely independent of TRPV1 signaling. Taken together, CNS effects of RTX originate dominantly in the peripheral disruption of TRPV1- signaling resulting in missing nociceptive thermal input to the CNS. No evidence of direct RTX-mediated disruption of brain network integrity–neither by peripheral nor by central excitotoxicity on TRPV1- expressing neurons- could be found.

### 5.3 Stimulus-driven brain networks & functional connectivity

Next, changes in interactivity of the affected brain regions were analyzed: using network based statistics (NBS) [51], changes in FC between functional groups of brain structures before (baseline) and after RTX administration were assessed (Fig 5).

Differences between baseline and RTX were found to be significant only for 50°C and higher, where RTX administration led to a massive decrease in FC between brainstem structures, superior colliculi, thalamic nuclei and hippocampus. Moreover, cortical (primary (S1) and secondary (S2) somatosensory cortex, association cortex (Ass), cingulate cortex (Cg)) connectivity was greatly attenuated. Astonishingly, thalamo-cortical and hippocampal-cortical connectivity remained largely unaffected, revealing two distinct subnetworks, linked via the basal ganglia (BG, especially striatum), that were involved in processing noxious temperatures at baseline and were subdued after desensitization of TRPV1-expressing neurons.

Solely, for the insular cortex to motor cortex (M), S1, Ass and the limbic output structures, an increased connectivity was found after RTX, but only for the highest temperature (Fig 5, 54°C).

Interestingly, part of the connectivity of the incoming brainstem structures seemed to stay intact after RTX-administration, which was a reinsurance, that the peripheral input was not completely circumvented by RTX: the proprioceptive and sensory in- and output from the posterior column–medial lemniscus pathway, medulla oblongata and the pontine nuclei remained intact.

**Fig 6:**
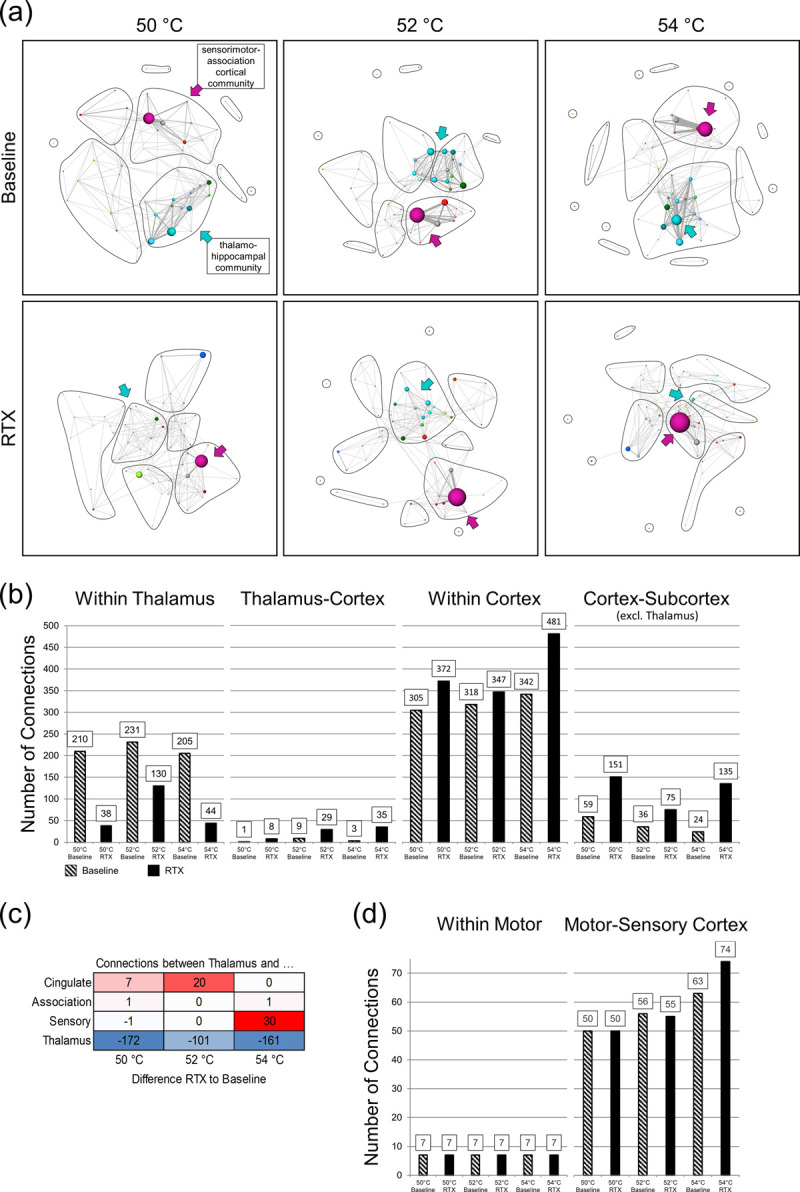
Stimulus-driven Blondel communities proofed that both affected subnetworks were an interconnected system. (a) RTX administration repressed mainly intra-thalamic connections (size of light blue nodes massively decreased, RTX) within the thalamo-hippocampal community (blue arrows) but enhanced the number of cortical connections (size of pink nodes increased, RTX) within the sensorimotor-association cortex community (pink arrows). Different functional groups are distinguished by the node colors (see legend in Fig 5), the size of the node represents the node degree (number of connections one functional group has to each other and also within the group). Edge thickness represents FC between two functional groups. Black lines encircle the Blondel communities. (b) RTX (solid) compared to baseline (shaded) reduced effectively the number of intra-thalamic (also see (c)) but increased all higher order connections, i.e. thalamo-cortical, within cortex as well as cortical-subcortical (excluding thalamus) connections. These can be observed for all stimulation temperatures, shown in the graphic are data from 50°C to 54°C. (c) Especially the number of connections between thalamus and cingulate cortex was increased for temperatures below 54°C whereas above 54°C no difference was found for thalamic-cingulate connectivity but instead connectivity between thalamus and sensory cortex increased. Shown is the difference between RTX and baseline for the number of connections between thalamus and the denoted regions. Marked in red are connections that were increased by RTX, blue are connections that were decreased by RTX. (d) Example of connections that are independent of RTX-administration: motoric output regions (comprising motor cortex and cerebellum) displayed stable connections within as well as to somatosensory cortex. (n_Baseline_ = 18; n_RTX_ = 9).

To evaluate how the structures of those subnetworks interacted with the remaining structures in more detail, Blondel communities were calculated for each group and temperature (Fig 6A). This analysis confirmed that the denoted structures of each subnetwork (subcortical vs. cortical) indeed were interacting rather with each other, than with structures outside their community. In addition, those structures were generally very well connected at baseline (Fig 6A, node size represents node degree, i.e. the number of connections one node has with others), underlining their importance for central thermal nociception.

Administration of RTX led on the one hand to a massive and significant reduction of thalamic connections (decrease of light blue node sizes), whereas on the other hand, the number of cortical connections (mainly sensory cortex, pink nodes) increased. The decrease of thalamic and increase of cortical connections was notable for all temperatures except 40°C, but only the highest temperatures are shown in Fig 6. Please not that here, all existing connections are taken into account in contrast to Fig 5 (only significant differences: cortical FC is decreased after RTX).

The total numbers of intra-thalamic and intra-cortical connections as well as connections between thalamus and cortex as well as cortex and subcortex (excluding thalamus) were calculated separately to further assess this effect (Fig 6B). The loss of TRPV1-signaling (RTX) reduced intra-thalamic and enhanced intra-cortical connections (sensory and association cortices) and subcortical-cortical. Also, the number of connections between thalamus and cortex increased after RTX: Here, we found a temperature-depending switch of thalamic connectivity (Fig 6C): the number of connections as well as functional connectivity between thalamus and particular the cingulate cortex was enhanced for temperatures that, after RTX, did not evoke brain responses to the heat stimulation (i.e. below 54°C). Connectivity between thalamus and sensory cortex was unchanged. In contrast, for 54°C, we found that connectivity between thalamus and cingulate cortex showed no difference to baseline. Instead, we found an increase in connectivity between thalamus and sensory cortex, which was accompanied by an increase in BOLD response amplitude and activated brain volume. Although many regions display changes in connectivity after RTX-administration, there are basic connections that remain stable, because they are not direct upstream targets of sensory and thalamic input. Exemplary, we show here connectivity within motor-associated regions (motor cortex and cerebellum), and between motor regions and sensory cortex (Fig 6D).

In order to disentangle if the found changes in connectivity were short-term phasic, stimulus-related effects or a tonic, general impact of RTX on brain circuitry, we investigated next the resting state connectivity as an independent measure.

### 5.4 Influence of peripheral TRPV1-signaling on RS-networks

**Fig 7:**
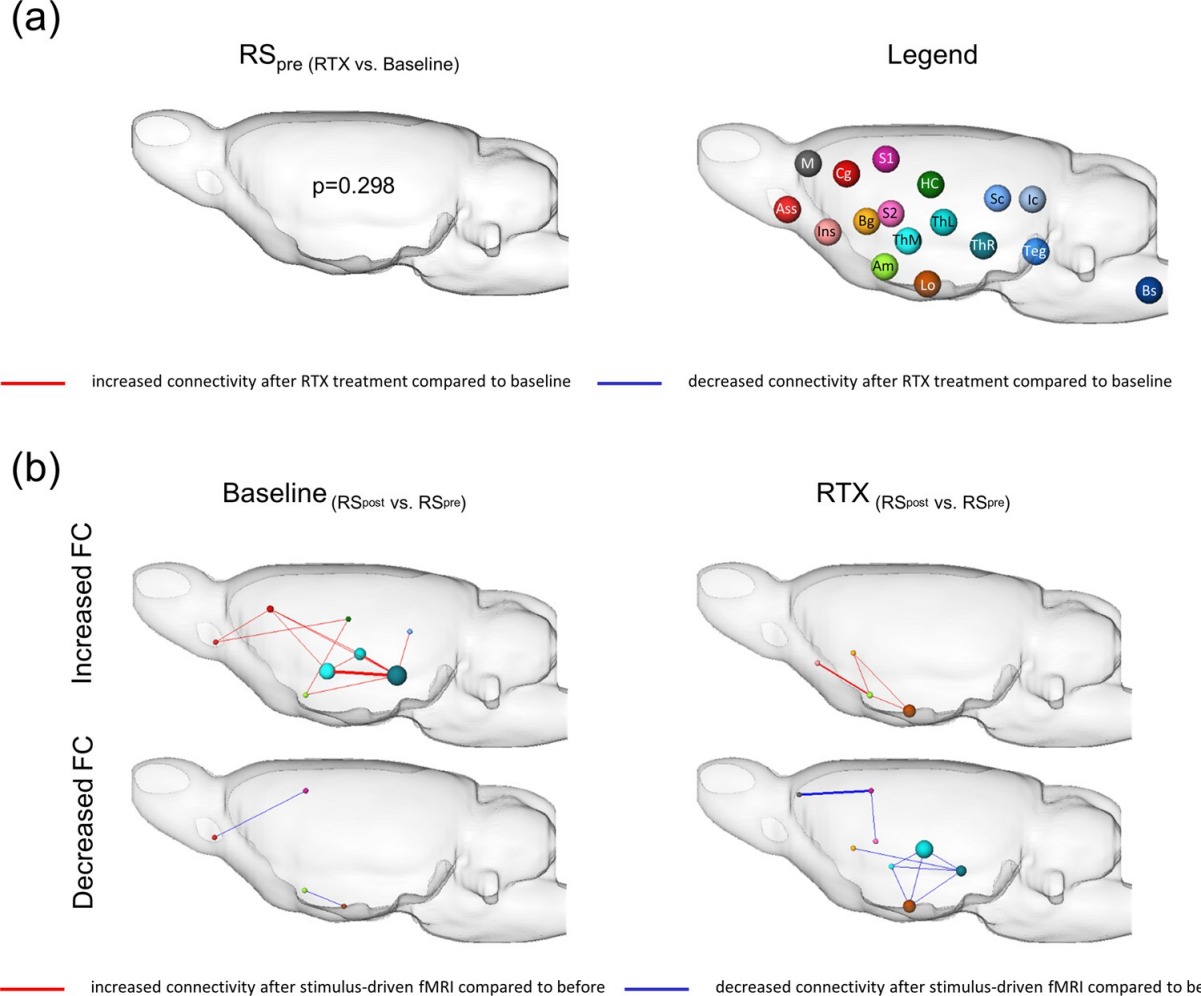
Impact of thermal stimuli on RS functional connectivity with and without peripheral TRPV1-signaling. No significant differences could be found between baseline and RTX RS-networks comparing the RS measurement that took place before the stimulus-driven stimulation ((a), RS_pre,_ p = 0.298). In contrast, the stimulation left significant imprints on RS-networks (RS_pre_ compared to RS_post_) for both groups (b). At baseline, animals showed distinct changes in RS FC after being subjected to the nociceptive stimulus-based fMRI session: increased FC (red edges) could be shown for thalamus, association cortex, amygdala and hippocampus, whereas reduced FC (blue edges) was found mainly in cortical and amygdala-hypothalamic regions. After RTX-treatment, enhanced FC could be found only in limbic regions (Lo, Am, BG, Ins). Reduced FC was now more prominent specifically in thalamus, but also found, alike baseline, in cortical and hypothalamic areas. Different functional groups are distinguished by the node colors. Node size represents number of significantly different connections each node has to others. Thickness of edges represents strength of connection (sum of Pearson r of all significantly differing connections). Statistical significance was calculated using NBS (a) and paired t-Test, permutation corrected p≤0.05 (b). (n_Baseline_ = 18; n_RTX_ = 9). Abbreviations: Am: amygdala; Ass: association cortex; BG: basalganglia; Bs: brainstem structures; Cg: cingulate cortex; HC: hippocampus; Ic: inferior colliculi; Ins: insular cortex; Lo: limbic output/hypothalamus; M: motor cortex; S1/S2: primary/secondary somatosensory cortex; Sc: superior colliculi; Teg: Tegmentum; thM/L: medial/lateral thalamus; ThR: other thalamic nuclei.

Two resting state measurements were conducted: first, directly before (RS_pre_) and second, after (RS_post_) the stimulus-driven fMRI sessions. In RS_pre_ (Fig 7A), no significant differences were found between baseline and RTX. It is known that the perception of peripheral sensory (noxious) stimuli alter various RS brain networks [49]. To assess how thermal nociception (baseline) or warmth sensation (RTX) imprints on the RS-networks, differences in FC were calculated between RS_pre_ and RS_post_ for both groups (Fig 7B). Usually, the extensive use of brain regions involved in nociceptive processing leaves a longer lasting imprint (in the range of 10^th^ of minutes) on the RS-networks, as shown for baseline, where FC of especially gating (thalamus) and evaluative (association cortex) structures were enhanced. Limbic (amygdala and hypothalamus) and perception-associated (sensory cortex) regions showed reduced FC. RTX treatment prevents perception of thermal nociception, but does not interfere with the perception of warmth. After RTX-treatment, enhanced FC was found within amygdala, basal ganglia and insular cortex, almost an opposing finding compared to baseline. Reduced FC was found mainly within thalamic regions and, alike baseline, within cortical and hypothalamic areas. Here, without nociceptive signaling, possible changes in FC may be attributed to the warmth stimuli or prolonged anesthesia and stress (constant scanner noise). Although, we cannot fully exclude that possible desensitization of TRPV1-neurons within the brain may also influence RS brain networks.

## 6. Discussion

### 6.1 Defining the threshold between warmth and nociceptive sensations

Although opening of TRPV1 channel pores starts at the temperature of 43°C in cell culture [55], the skin surface threshold for nocifensive behavior is considerably higher. Studies, where the hairy backside of paws was irradiated, reported flinching at 45–46°C and flight reactions at 51–52°C [56]. This fits to our finding, that the lowest stimulation temperature allowing to detect stimulus-evoked fMRI responses was 48°C, i.e. the threshold for fMRI responses between warmth and nociceptive sensation for anesthetized rats with contact heat at the dorsal hind paw.

### 6.2 Resiniferatoxin reduced effectively the central response to noxious heat

According to our results, desensitizing TRPV1-expressing neurons was successful: RTX treatment prevented any significant increase of BOLD response amplitude and activated brain volume at temperatures above the noxious threshold. In contrast, at baseline, both parameters correlated strongly with temperature increase (1.5% increase in response amplitude, around 6-fold increase of activated volume for 54°C). Therefore, this group comparison denotes the peripheral TRPV1-mediated discrimination between warmth and noxious heat.

There are many thermal transducer channels besides TRPV1, located on C- and Aδ-fibers, initially activated around 25–35°C and still active in noxious ranges: TRPV3, TRPV4 or KCNK2 (review [57]). Nonetheless, in RTX-treated animals, no significant activation increase was found for stimulus temperatures below 54°C. Interestingly, while desensitization of TRPV1-expressing neurons abolishes behavioral responses to noxious heat [58], TRPV1-knockout mice show only minor deficits in thermoception [59]. Additional knockout of TRPA1 and TRPM3 is necessary but sufficient to preclude perception of noxious heat completely [60]. Therefore, our finding of the broad diminishment of global brain responses indicates that TRPV1-expressing fibers are almost solely responsible for peripheral heat perception, and that there seems to be no parallel fiber population expressing other heat transducer channels besides TRPV1. We therefore functionally validated that C- and Aδ-fibers don’t express solely one type of transducer channel: for TRPV1, co-localization of other TRP-family members (e.g. TRPM8, TRPA1 [9]), voltage-gated potassium channels (e.g. K_v_1.4 [61]), and cannabinoid receptors (CB1 [61]) is known. As consequence, after RTX, also those co-expressed channels are lost. Therefore, desensitization of TRPV1-expressing fibers using RTX might influence perception of other sensations, interaction and even modulation thereof (CB1).

Even with the highest temperature (54°C), response amplitudes and activated brain volumes of the RTX-treated animals were much lower than at baseline (cf. activated volume: S2 Fig; response amplitude: Fig 4), indicating either that the stimulus is not perceived as noxious heat but still as warmth or that few TRPV1-expressing neurons survived the treatment, as was previously discussed by Mitchell *et al*. [32].

### 6.3 Brain regions involved in TRPV1-mediated thermo-sensation

The major CNS target for sensory information is the thalamus, functionally divided into lateral (sensory) and medial (affective/motivational) parts [5]. From the thalamus, information is distributed into many cortical and limbic brain structures (see [62] for detailed review). At baseline, a significant increase in response amplitude and activated brain volume was found in most nociception-associated brain structures for temperatures above 48°C, upstream of lateral and medial thalamus. In contrary, no such corresponding temperature-dependent rise of stimulus-response curves could be found after RTX.

After RTX, a few brain regions, such as cingulate and insular cortex, showed a significant signal rise only at 54°C, even though merely one third of baseline responses. Cingulate and insular cortices are part of the central execution network and fulfil cognitive as well as emotion-related tasks [63, 64], evaluating aversion of stimuli and integrating them with previous experience and context. They play an important role in cognition-modulated descending pain control, e.g. thermal stimuli rated less painful when a person is forced to concentrate on specific tasks [65–67]. The fact, that both regions showed an increase in BOLD response amplitude after RTX with highest temperature, indicates that at least to some extent the most noxious stimulus impacted on them even after desensitization of TRPV1-expressing neurons. This may imply that heat stimuli may be considered more salient, unpleasant and aversive with increasing stimulation temperature.

Taken together, our classical BOLD results indicate that RTX-administration diminished the peripheral input to thalamic nuclei. This confirmed our basic hypothesis, that dysfunction of TRPV1-expressing nociceptive peripheral C- and Aδ-fibers disrupted higher order heat nociception. The general loss of thalamic input did not trigger any substantial upstream activation within the lateral-discriminative and medial-emotional nociceptive systems.

Classical BOLD-signal analyses demonstrated an extensive suppression of the overall brain activity, which affected almost every brain region. To gain a more comprehensive insight, the interaction between those brain regions was investigated using graph-theoretical analyses based on the FC between brain regions.

### 6.4 Graph theory of stimulus driven BOLD responses revealed the importance of two distinct brain subnetworks involved in thermal nociception

Interestingly, removing peripheral nociceptive thermal input did lead to a general decrease of whole-brain FC. However, the overall information flow efficacy, reflected e.g. by the small world index, was not influenced.

Graph theory could separate two distinct subnetworks showing diminished FC after RTX. The first, subcortical subnetwork consisted of thalamus, hippocampus, brainstem, superior colliculi and included basal ganglia, amygdala and limbic output structures such as hypothalamus and PAG, with the latter being a central part of the descending pain modulating pathway. The RTX main effect, reflected by a massive FC decrease, could be found within thalamic, thalamo-hippocampal and brainstem connections. The second, cortical subnetwork included primary sensory, motor and association cortices. These two subnetworks were linked via the limbic system, especially via amygdala and basal ganglia, likely through the ventral amygdalofugal pathway which projects from amygdala to olfactory nucleus, piriform cortex, association cortex (especially cingulate cortex) and striatum (nucleus accumbens and caudate putamen) [68]. The amygdalar projections to the basal ganglia are part of the basal ganglia circuit relevant for stimulus-response associative learning [69].

Compared to baseline, RTX-treated rats showed enhanced connectivity between thalamus and cortex, cingulate cortex in particular (cf. Fig 6C), for temperatures between 50 and 54°C. Conversely, normal thalamo-cingulate connectivity marks perception of noxious thermal stimulation at baseline. For 54°C, this difference vanishes. Assuming that 54°C is experienced as noxious in both groups, this indicates that noxious heat stimuli, independent from their perceived saliency or intensity, disrupt thalamo-cingulate connectivity. This is concordant with findings, that the cingulate cortex, specifically its functional interaction with the posterior thalamus, is involved in attentional modulation of pain [67]. Interestingly, while intra-thalamic connectivity is decreased by RTX, thalamo-cortical, intra-cortical and cortical-subcortical connectivity is enhanced. Previous work has shown the importance of thalamo-cortical-striatal loops during action-selection in dependence on stimulus saliency [70]. Possibly, decoupling of thalamo-cortical-striatal loops due to the loss of highly salient peripheral input from thalamus after RTX may lead to the denoted enhancement of cortical connectivity.

### 6.5 RTX impact on RS-networks

RS-network analyses revealed that nociceptive stimulation leaves specific footprints regarding functional connectivity [52]. First, no significant differences in RS_pre_-networks could be detected between the groups before and after RTX treatment. This implicates that the sustained desensitization of TRPV1-expressing nociceptors had no profound impact on basic RS-networks. Second, comparing RS-networks before and after stimulus-presentation, enhanced FC was found for baseline in regions linked to memory formation and emotion regulation (hippocampus, cingulate and association cortex, amygdala). This might reflect a trace of the perceived unpleasantness, indicating ongoing cognitive processing potentially involving memory formation. After RTX-treatment, enhanced FC could be found within the amygdala, indicating that basic aversive networks remain activated, probably due to the scanning situation. We cannot exclude that possible blunting of central TRPV1-expessing neurons may contribute additionally. Although, if that would be the case, one would expect imprints in basic resting state networks. In particular, when comparing two RS networks separated by a nociceptive stimulation, it should also be considered that the second RS (here, RS_post_) might be more affected by prolonged anesthesia and that nociceptive stimulation might lead to modulatory effects of the noradrenergic/orexine system originating from the locus coeruleus [71, 72]. Nevertheless, we found the significant RS-network differences at baseline and after RTX using the very same experimental setting.

### 6.6 Clinical relevance, critical notions and outlook

Despite some undesirable side effects such as hypothermia and increased risk of burns due to impaired heat perception [73], the future potential use of TRPV1 agonists for treatment of pain is a hot topic in research. Local use of capsaicin, e.g. 8% capsaicin patches [74], showed good efficacy in the usually difficult treatment of neuropathic pain. Of note, RTX is already used as an analgesic for dogs with cancer and arthritic pain [31].

Regarding the limitations of our study, a common confounding factor in the measurement of cerebral activity in animals is anesthesia. In humans, isoflurane is known to modulate dose-dependently cerebral blood flow and glucose metabolism [75, 76]. Compared to other anesthetics, isoflurane is weakly analgesic and least impacting on basic physiology [77]. RS-BOLD fluctuations are maintained albeit decreased under isoflurane [78], which is also true for thalamo-cortical connectivity [79]. Because all measurements, baseline and RTX, were conducted with the identical anesthetic protocol, valid conclusions about RTX effects can still be drawn. However, the modulatory effects of isoflurane on the brain connectivity have to be taken into account carefully, particular for generalization to the awake, e.g. human situation.

For the scope of this study, only male rats were used, mainly to limit animal numbers (twice the number would have to be used when assessing both genders) and to circumvent the confounder of the estrus cycle in females. Knowing that there are gender-related differences [80] in nociception in humans and animals and that these differences are associated with TRPV1, our findings may not be fully applicable to females [81, 82].

As stated above, RTX injections can be accompanied by major stress. For the sake of the 3R-principle, and as there are yet sufficient publications covering behavioral tests under RTX-treatment [23, 27, 32, 83], we abstained deliberately from repeating behavioral tests, keeping stress levels and experimental time for the rats as minimal as possible. Therefore, we do not provide repetitive behavioral data on successful desensitization of TRPV1-expressing neurons.

Stress may also modulate brain FC, considering that pain is also a strong stressor. Therefore, it may seem difficult to attribute the findings strictly to (the RTX-mediated loss of) nociception. Of note, TRPV1-expressig fibers are mainly desensitized [84] already after the first RTX injection, performed under anesthesia, and RTX is also used in veterinary medicine [34]. Therefore we assume, that there is no major increased stress-level directly due to the RTX injection or injection-associated pain itself. Stimulus-driven FC is more robust to tonic confounders such as stress, as only strictly stimulus-evoked signals are taken into account, but RS FC was previously shown to be readily impacted by stress [85]. The enhanced mucus secretion after RTX injection induces stress in the animals and may therefore impact especially on RS FC. However, no differences in RS_pre_ FC were observed between baseline and RTX groups, suggesting that treatment or associated stress did not alter brain FC at rest.

A future extension would be the introduction of a positive control by means of a different, e.g. mechanical, stimulation to verify that RTX treatment solely diminishes thermal nociceptive inputs.

Applying topical capsaicin, a potent activator of TRPV1, could serve as an *in vivo* functional control for successful desensitization of TRPV1-expressing neurons.

### 6.7 Summary

Imaging rats before and after RTX-desensitization of TRPV1-expressing neurons allowed detailed insight into CNS circuitry of processing innocuous and noxious thermal stimuli: after RTX, the rats ceased to show any temperature-dependent rise in brain activity to temperatures between 48 and 52°C. Only for highly noxious 54°C, slight responses could be found. Graph-theoretical analyses revealed, that even though during the basic resting state and during stimulation the overall network efficacy was not altered, two distinct brain subnetworks processing noxious input were affected by RTX: one subcortical (brainstem, thalamus, hippocampus, basal ganglia, and amygdala), and one cortical (primary sensory, motor and association cortices), being linked by limbic system and basal ganglia. Furthermore, network analyses discovered a RTX-induced disruption of intra-thalamic connectivity, followed by enhanced upstream connectivity, highlighting the importance of thalamo-cortical-striatal loops for central nociceptive processing in the normal brain.

## Supporting information

S1 Fig Pearson correlation between BOLD response amplitude and the linear temperature characteristic curve. Marked in red are correlations r>0.8. At baseline, we found that activity paralleled the increase in temperature very well in most structures, while no correlation was found after RTX. (n_Baseline_ = 18; n_RTX_ = 9).
(TIF)

S2 Fig Mean activated brain volume for representative functional groups. Similar to the BOLD response amplitude, desensitization of TRPV1-expressing neurons also reduced the activated brain volume in most brain regions. Peripheral paw stimulation with temperatures above 48°C led to no further increase in most regions. This effect was found only for regions associated with nociceptive processing, as regions involved in basal homeostasis tasks such as brainstem and some midbrain regions were unaffected. Statistical significance between groups was calculated using homoscedastic Student’s t-test and corrected for multiple comparisons by FDR q = 0.05. Data are represented as mean ± standard error (SEM). * p≤0.05; ** p≤0.01; *** p≤0.001. (n_Baseline_ = 18; n_RTX_ = 9).
(TIF)

S3 Fig Brain areas involved in nociceptive processing in the rat. Nociceptive information from the periphery is conducted via the spinothalamic tract to the thalamus, where it is filtered and forwarded to higher order brain structures. The lateral thalamus projects into mainly sensory-descriptive cortical layers, whereas the medial thalamus projects mainly to emotional-affective structures, such as the limbic system. The descending pathways relay anti-nociceptive signals to hypothalamus, raphe nucleus and periaqueductal grey, modulating the nociceptive input from the dorsal horn neurons. Adapted from (Sergeeva et al., 2015). Abbreviations: Am: amygdala; Ass: association cortex; BG: basalganglia; Cer: cerebellum; HC: hippocampus; Hy: hypothalamus; Ins: insular cortex; M: motor cortex; S1/S2: primary/secondary somatosensory cortex; thM/L: medial/lateral thalamus; PAG: periaqueductal grey.
(TIF)

S4 Fig Characterization of brain network efficiency. No significant difference between baseline and RTX was found for the normalized cluster-coefficient γ (a) or the normalized path length λ (here only 40°C significant) (b). The path length of both groups increased slightly with rising temperatures, indicating a decreased efficacy in information processing of noxious temperatures, which was noted also as a decreasing small world index σ (c). Desensitization of TRPV1-expressing neurons (RTX) had no effect on the global efficacy of information processing within the rat brain: efficacy of information flow (small world index σ) was negatively correlated with the applied temperature and this was independent of the abundance of TRPV1 as a similar effect was found in both groups. Statistical significance between groups was calculated using homoscedastic Student’s t-test and corrected for multiple comparisons by FDR q = 0.05. Data are represented as mean ± standard error (SEM). * p≤0.05; ** p≤0.01; *** p≤0.001. (n_Baseline_ = 18; n_RTX_ = 9).
(TIF)

S1 Data Data underlying Figs 2 and 4, S2 and S4 Figs. (XLSX)
