# Supplementary figures and images for: Imaging the influence of peripheral TRPV1-signaling on cerebral nociceptive processing applying fMRI-based graph theory in a resiniferatoxin rat model
Source: PLoS One. 2022 Apr 28;17(4):e0266669. doi: 10.1371/journal.pone.0266669 (PMC9049522; doi:10.1371/journal.pone.0266669)

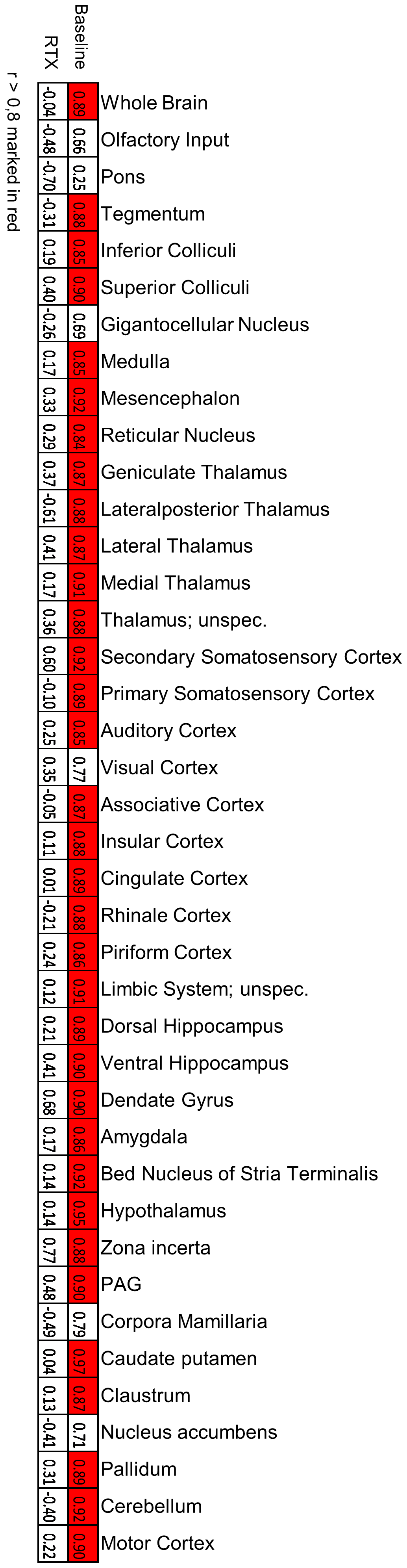

Supplement: S1 Fig — Marked in red are correlations r>0.8. At baseline, we found that activity paralleled the increase in temperature very well in most structures, while no correlation was found after RTX. (nBaseline = 18; nRTX = 9). (TIF) [file pone.0266669.s001.tif]

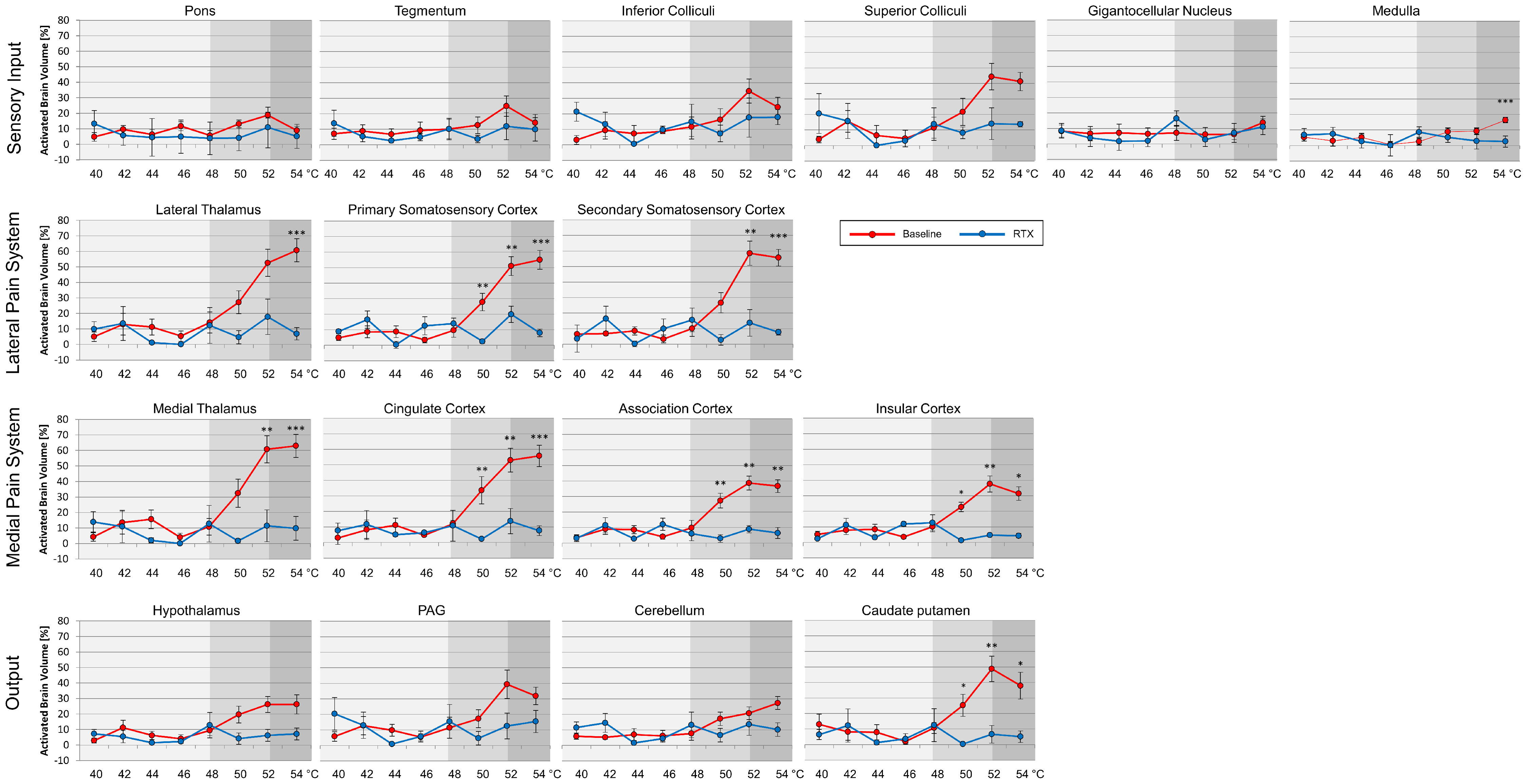

Supplement: S2 Fig — Similar to the BOLD response amplitude, desensitization of TRPV1-expressing neurons also reduced the activated brain volume in most brain regions. Peripheral paw stimulation with temperatures above 48°C led to no further increase in most regions. This effect was found only for regions associated with nociceptive processing, as regions involved in basal homeostasis tasks such as brainstem and some midbrain regions were unaffected. Statistical significance between groups was calculated using homoscedastic Student’s t-test and corrected for multiple comparisons by FDR q = 0.05. Data are represented as mean ± standard error (SEM). * p≤0.05; ** p≤0.01; *** p≤0.001. (nBaseline = 18; nRTX = 9). (TIF) [file pone.0266669.s002.tif]

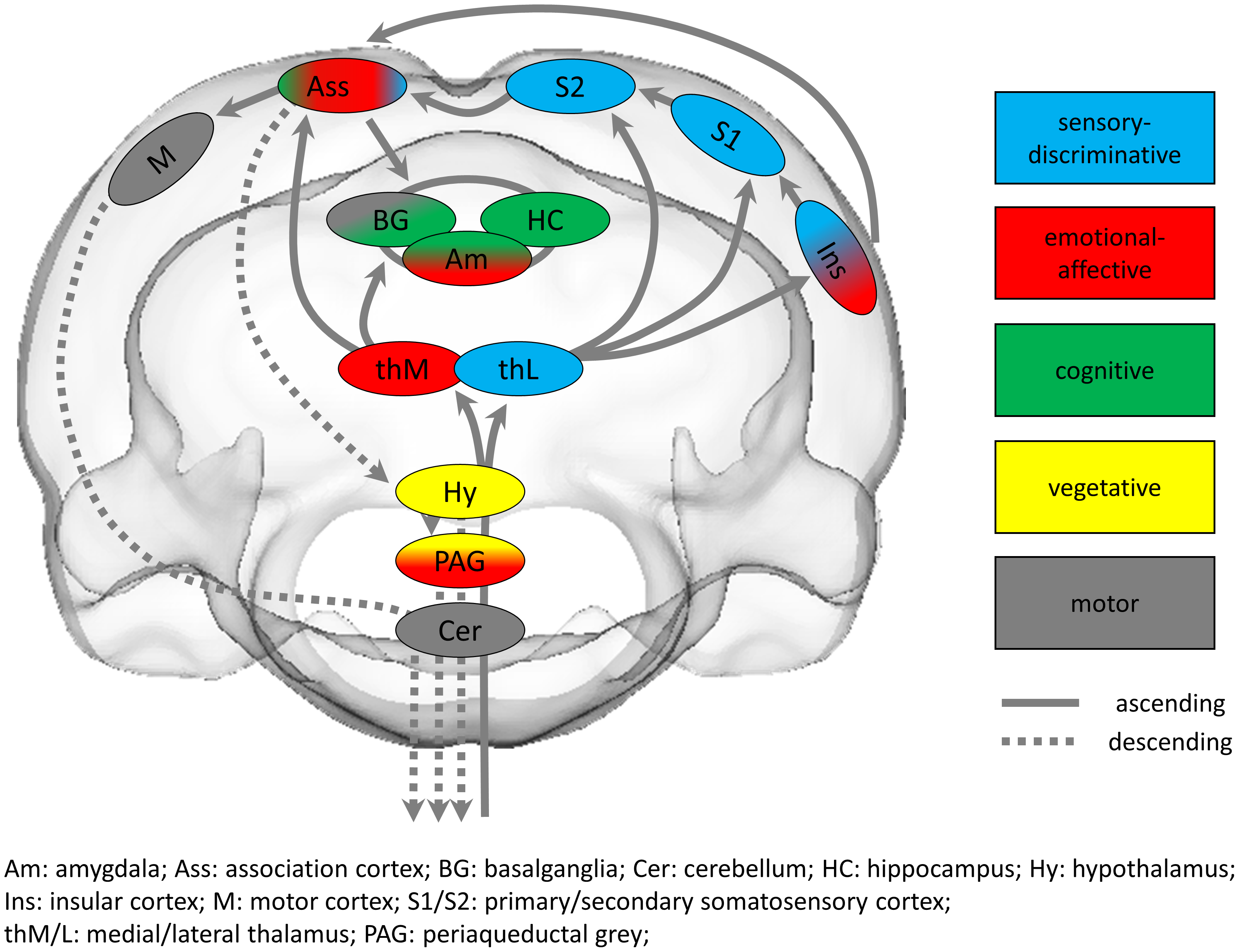

Supplement: S3 Fig — Nociceptive information from the periphery is conducted via the spinothalamic tract to the thalamus, where it is filtered and forwarded to higher order brain structures. The lateral thalamus projects into mainly sensory-descriptive cortical layers, whereas the medial thalamus projects mainly to emotional-affective structures, such as the limbic system. The descending pathways relay anti-nociceptive signals to hypothalamus, raphe nucleus and periaqueductal grey, modulating the nociceptive input from the dorsal horn neurons. Adapted from (Sergeeva et al., 2015). Abbreviations: Am: amygdala; Ass: association cortex; BG: basalganglia; Cer: cerebellum; HC: hippocampus; Hy: hypothalamus; Ins: insular cortex; M: motor cortex; S1/S2: primary/secondary somatosensory cortex; thM/L: medial/lateral thalamus; PAG: periaqueductal grey. (TIF) [file pone.0266669.s003.tif]

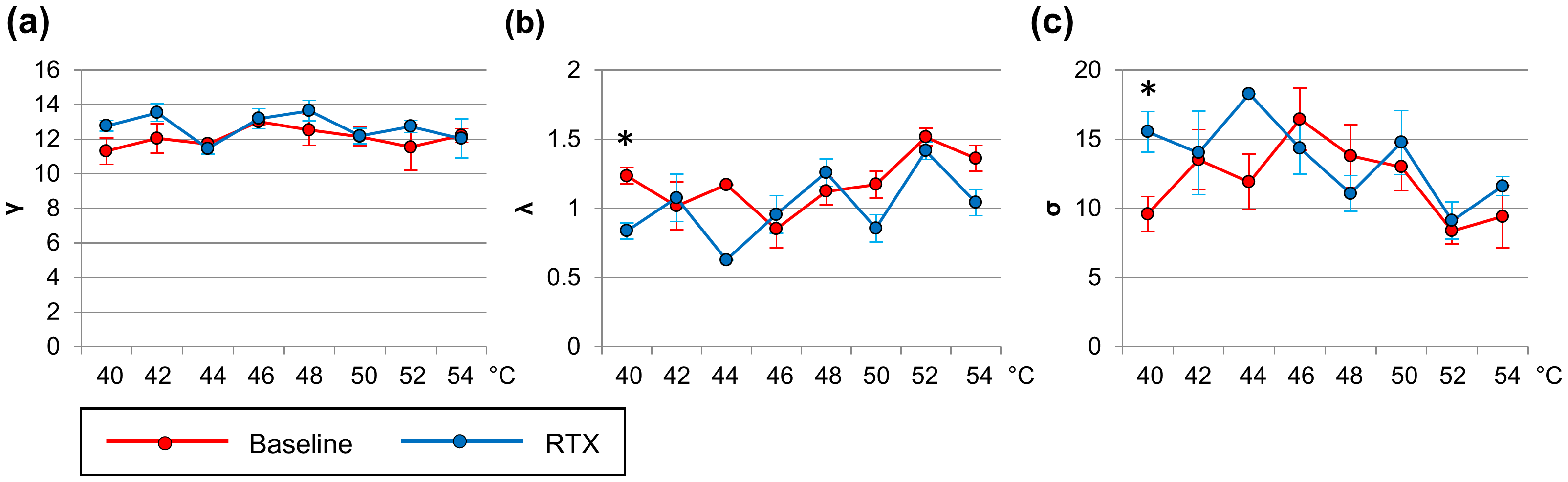

Supplement: S4 Fig — No significant difference between baseline and RTX was found for the normalized cluster-coefficient γ (a) or the normalized path length λ (here only 40°C significant) (b). The path length of both groups increased slightly with rising temperatures, indicating a decreased efficacy in information processing of noxious temperatures, which was noted also as a decreasing small world index σ (c). Desensitization of TRPV1-expressing neurons (RTX) had no effect on the global efficacy of information processing within the rat brain: efficacy of information flow (small world index σ) was negatively correlated with the applied temperature and this was independent of the abundance of TRPV1 as a similar effect was found in both groups. Statistical significance between groups was calculated using homoscedastic Student’s t-test and corrected for multiple comparisons by FDR q = 0.05. Data are represented as mean ± standard error (SEM). * p≤0.05; ** p≤0.01; *** p≤0.001. (nBaseline = 18; nRTX = 9). (TIF) [file pone.0266669.s004.tif]
